# The Impact of L-Dex^®^ Measurements in Assessing Breast Cancer-Related Lymphedema as Part of Routine Clinical Practice

**DOI:** 10.3389/fonc.2016.00192

**Published:** 2016-09-05

**Authors:** Alison Laidley, Beth Anglin

**Affiliations:** ^1^Texas Oncology, Texas Breast Specialists, Dallas, TX, USA; ^2^North Texas Surgical Oncology Associates, Plano, TX, USA

**Keywords:** breast cancer, lymphedema, bioimpedance, SLNB, ALND, BCRL, BIS, L-Dex

## Abstract

**Purpose:**

With improved survivorship, the prevalence of breast cancer-related lymphedema (BCRL) continues to increase, leading to impairment of a patients’ quality of life. While traditional diagnostic methods are limited by an inability to detect BCRL until clinically apparent, bioimpedance spectroscopy (BIS) has been shown to detect subclinical BCRL. The purpose of this study is to evaluate the role of BIS in the early detection of BCRL, as well as assessment of response to BCRL treatment.

**Methods:**

A retrospective review of 1,133 patients treated between November 2008 and July 2013 at two surgical practices was performed. Eligible patients (*n* = 326) underwent preoperative and postoperative L-Dex measurements. Patients were identified as having subclinical lymphedema if they were asymptomatic and the L-Dex score increased >10 U above baseline and were monitored following treatment. Patients were stratified by lymph node dissection technique [sentinel lymph node biopsy (SLNB) vs. axillary lymph node dissection (ALND)] and receipt of BCRL treatment.

**Results:**

The average age of the cohort was 56.2 years old, and mean follow-up was 21.7 months. Of the 326 patients, 210 underwent SLNB and 116 underwent ALND. BCRL was identified by L-Dex in 40 patients (12.3%). The cumulative incidence rate of subclinical lymphedema was 4.3% for SLNB (*n* = 9) and 26.7% for ALND (*n* = 31). Of those diagnosed with BCRL, 50% resolved following treatment, 27.5% underwent treatment without resolution, and 22.5% had resolution without treatment. The prevalence of persistent, clinical BCRL was 0.5% for SLNB and 8.6% for ALND.

**Conclusion:**

This study demonstrates both the feasibility and clinical utility of implementing L-Dex measurements in routine breast cancer care. L-Dex identified patients with possible subclinical BCRL and allowed for assessment of response to therapy.

## Introduction

Breast cancer represents the most common non-cutaneous cancer among women and as such, treatment paradigms and survivorship strategies continue to evolve (1). Over the past several decades, survival for all stages of breast cancer has improved, leading to more long-term survivors, and therefore a greater prevalence of chronic sequelae of treatment (2). One complication that can significantly impair quality of life and has been increasingly studied is breast cancer-related lymphedema (BCRL). BCRL develops due to impaired drainage function of the lymphatic system and is progressive, chronic, costly, and frequently an emotionally devastating sequelae of breast cancer treatment (3, 4).

**Figure 1:**
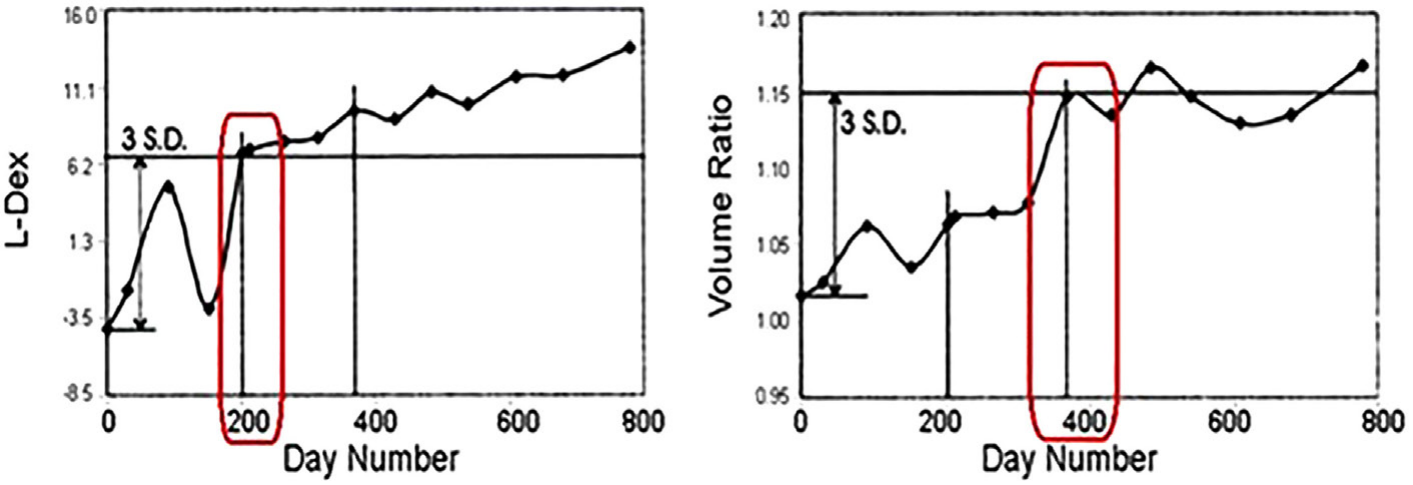
**Identification of subclinical lymphedema with L-Dex prior to significant volume increase [adapted from Cornish et al. (18)]**.

Incidence rates from BCRL vary widely with rates of 0–94% reported in the literature (Table 1) based on extent and modality of locoregional and systemic therapies, diagnostic techniques utilized, and duration of follow-up (5–16). Traditional methods to diagnose BCRL include techniques that assess the entire volume of the limb, such as circumference measurements, water displacement, and patient self-report. These techniques are limited because they require BCRL to be clinically apparent before detection, thus lacking sensitivity to detect small changes in extracellular fluid (ECF) and subclinical BCRL (5). However, the pathophysiology of BCRL suggests that the ECF compartment is the most relevant area of concern and studies show it is this compartment that changes during the early stages of BCRL. Therefore, assessments of the ECF would potentially allow for the detection of subclinical BCRL at an earlier time than traditional diagnostic techniques that assess the total volume of the limb (17, 18). Bioimpedance spectroscopy (BIS) is a technique that assesses the ECF compartment and therefore allows for the subclinical detection of BCRL when visible swelling is not apparent. Studies establishing the feasibility of BIS in assessing BCRL have demonstrated an earlier time to diagnosis than traditional diagnostic modalities (18–20). L-Dex is the score reported by the L-Dex U400 unit (ImpediMed Limited, Australia) and represents the ECF ratio of the at-risk (or affected) limb to the unaffected limb. As ECF accumulates in the at-risk limb, the L-Dex score increases. It is therefore a sensitive tool for assessing early accumulation of ECF and has demonstrated the ability to predict the onset of lymphedema up to 10 months prior to clinical diagnosis (18). Figure 1 shows published patient data for simultaneous L-Dex score and inter-limb volume ratio (from circumferential tape measurements) in which the L-Dex score increases to a subclinical level approximately 200 days prior to the volume ratio (18).

**Table 1:** **Incidence rates reported in the literature**.

Reference	Diagnostic method	Duration of follow-up	Subjects	Incidence rates	
Haid et al. (7)	Self-report Inter-limb circumference (tape) >2 cm Clinical examination	ALND mean 25 months SLNB mean 18 months	ALND 140 SLNB 57	ALND 27.1% SLNB 3.5%	
Veronesi et al. (8)	Inter-limb circumference (tape) >2 cm	24 months	ALND 100 SLNB 100	ALND	6 months 8% 24 months 12%
				SLNB	6 months 0% 24 months 0%
Armer et al. (9)	Inter-limb circumference (tape) >2 cm Self-report	Mean 28 months	102	SLNB 22.2% ALND 43.3%	
Leidenius et al. (10)	Self-report Limb circumference Clinical examination	36 months	ALND 57 SLNB 92	ALND	Clinical 13% Self-report 28%
				SLNB	Clinical 1% Self-report 5%
Clark et al. (11)	Inter-limb volume (tape) >5%	36 months	188	20.7%	
Francis et al. (12)	Inter-limb volume and/or circumference (tape) >5%	12 months	155	ALND 47.1% SLNB 16.8%	
Langer et al. (13)	Inter-limb circumference (tape) >2 cm Self-report	ALND mean 29.5 months	659	ALND 19.1%	
		SLNB mean 31.0 months		SLNB 3.5%	
Hayes et al. (14)	BIS > 3 SD	18 months	287	Point	6 months 10.7% 12 months 8.0% 18 months 14.9%
				Cum.	6 months 10.7% 12 months 22.7% 18 months 33.6%
Stout Gergich et al. (15)	Limb volume (perometry) >3% change from baseline	18 months	196	21.9%	
Johansson and Branje (16)	Limb volume (water disp) >5% change from baseline	12 months	292	ALND with radiation 38.7%	
Armer and Stewart (6)	Limb circumference (tape) >2 cm from baseline and/or contralateral limb Limb volume (perometry) >200 ml from baseline and/or contralateral limb Limb volume (perometry) >10% from baseline and/or contralateral limb Self-report	60 months	236	12 months 66, 40, 22, and 32% 24 months 81, 56, 36, and 35% 36 months 88, 66, 43, and 39% 60 months 94, 83, 55, and 43%	

At this time, the importance of early detection and subsequent early intervention has been demonstrated with the publication of a prospective trial as well as several other studies demonstrating improvements in patient outcomes with intervention at earlier phases of BCRL (15, 21–26). In 2015, the National Comprehensive Cancer Network updated their survivorship guidelines to reflect this changing paradigm by noting that part of post-treatment follow-up for breast cancer is to “educate, monitor, and refer for lymphedema management (27).” L-Dex, by its ability to detect subclinical BCRL, represents an innovative strategy for breast cancer programs to meet this evidence-based guideline. As such, the purpose of this study is to evaluate the clinical utility of BIS to detect and monitor the early development of BCRL and assessment following BCRL treatment in a large cohort of patients evaluated preoperatively and as part of long-term breast cancer follow-up.

## Materials and Methods

A retrospective chart review of 1,133 breast cancer patients treated at two surgical practices between November 2008 and July 2013 was performed with Institutional Review Board approval given by the Western Institutional Review Board. Inclusion criteria included (1) some form of axillary staging [sentinel lymph node biopsy (SLNB) or axillary lymph node dissection (ALND)], (2) preoperative L-Dex assessment, and (3) a minimum of two subsequent L-Dex assessments. Exclusion criteria included (1) bilateral axillary surgery, (2) previously documented diagnosis of BCRL, (3) pregnancy, and (4) implanted electronic cardiac device, such as a pacemaker. A total of 326 patients meeting such criteria were identified and represent the cohort upon which the analysis was performed. Surgical technique, axillary sampling technique, and L-Dex assessments were available but body mass index (BMI), utilization of systemic and radiation therapy was not.

**Figure 2:**
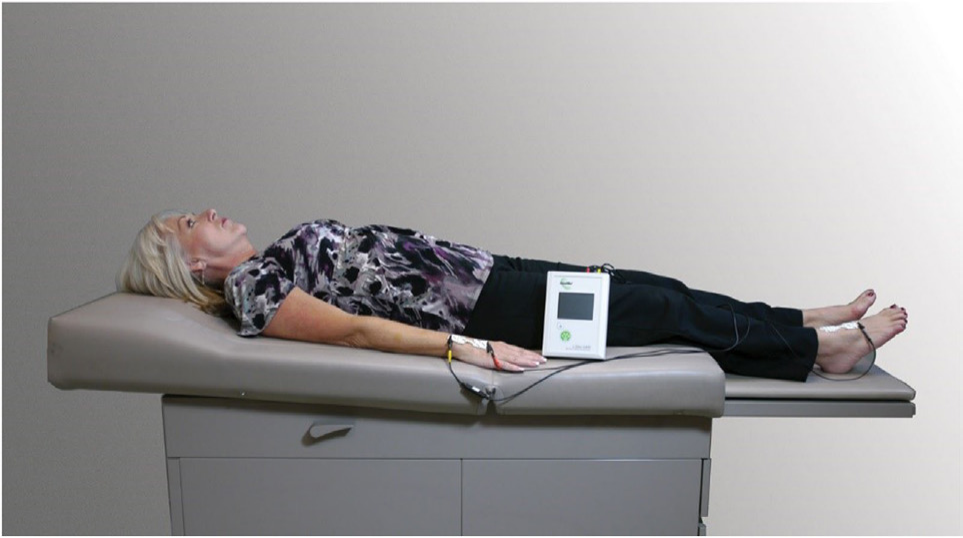
**Patient undergoing L-Dex U400 measurement**.

With regard to assessment technique, L-Dex readings were taken using the L-Dex U400. Measurements were taken with patients lying supine on a non-metallic surface utilizing a standardized technique as demonstrated in Figure 2 (19). Electrodes were placed on the skin on the midline dorsal surface of the wrist at the level of the ulnar styloid process and on the skin on the midline anterior surface of the ankle at the level of the medial and lateral malleolus bones (9). The L-Dex represents the ratio of measured impedance between the at-risk limb and a control limb as compared to an equivalent healthy population. The linearized L-Dex score allows BIS results to be compared across gender, limb dominance, and at-risk limb. When a preoperative healthy baseline value has been measured, a change in >10 L-Dex units is indicative of the presence of subclinical lymphedema and is equivalent to a change in >3 SD of the healthy population (18).

Patients were prospectively monitored preoperatively and assessed every 3 months within the first 2 years after surgery, in accordance with recommendations from Stout Gergich et al. (15), unless recommended otherwise, such as in the case of positive assessment for lymphedema. At the first presentation of subclinical lymphedema, patients were treated using traditional methods (compression sleeve, massage, and/or physical therapy) as determined by the practicing physician. Resolution of BCRL was defined as a return to within 10 L-Dex units of the preoperative assessment at the end of follow-up.

## Results

A total of 326 women met the selection criteria and were evaluated for this study. The mean age of the cohort was 56.2 years old with a mean time from preoperative L-Dex assessment to first postoperative assessment of 5.4 months. Mean overall follow-up was 21.7 months (range: 3.7–54.0 months). For the entire cohort, 155 patients (47.5%) underwent lumpectomy and 171 (52.5%) underwent mastectomy. Two hundred ten patients underwent SLNB (64.4%, mean 2.8 nodes) and 116 ALND (35.6%, mean 12.2 nodes). Table 2 presents patient characteristics, surgical technique, and follow-up time.

**Table 2:** **Patient characteristics**.

	Total	SLNB	ALND
Total number of subjects	326	210	116
Lumpectomy	155	123	32
Mastectomy	171	87	84
Mean age at baseline (years μ ± σ)	56.2 ± 11.1	55.6	57.5
Mean time to first follow-up (months μ ± σ)	5.4 ± 3.7	5.3	5.7
Mean total follow-up time (months μ ± σ)	21.7 ± 12.2	21.2	22.7

Breast cancer-related lymphedema status based on L-Dex score, treatment, and outcomes, stratified by axillary surgery are displayed in Table 3. Of the 326 patients evaluated, the cumulative incidence of subclinical BCRL was 12.3% (*n* = 40) with a rate of 4.3% (*n* = 9) for those undergoing SLNB and 26.7% (*n* = 31) for those undergoing ALND. For those undergoing SLNB, the mean time to positive L-Dex assessment was 5.6 months (2.3–13.6 months) from preoperative assessment and mean follow-up from subclinical BCRL diagnosis of 15.7 months (0.6–43.7 months). For those undergoing ALND, the mean time to positive assessment was 7.5 months (1.3–26.1 months) from preoperative assessment and mean follow-up from subclinical BCRL diagnosis of 13.4 months (0–18.1 months). Among the 286 patients not diagnosed with BCRL, 30 patients used prophylactic garments when performing perceived high risk activities, such as air travel with no patient progressing to BCRL.

**Table 3:** **Patient response to treatment based on L-Dex score**.

Assessment based on L-Dex	Treatment	Outcome	Total	SLNB	ALND
**Normal L-Dex values**					
Normal			256	184	72
Normal (L-Dex trending up)	Yes	Remain normal	30	17	13
Totals for normal			286	201	85
**Elevated L-Dex values**					
BCRL	Yes	Resolved	20	6	14
BCRL	Yes	Unresolved	11	1	10
BCRL	No	Resolved	9	2	7
Totals for BCRL			40	9	31
Total number of subjects			326	210	116
Cumulative incidence of lymphedema (%)			12.3	4.3	26.7

Of the 31 patients diagnosed with BCRL who underwent treatment (ALND = 24, SLNB = 7), 20 (64.5%) had resolution of their BCRL at the end of the study. Nine patients (ALND = 7, SLNB = 2) diagnosed with BCRL did not undergo treatment with all demonstrating resolution of their BCRL. Seventeen SLNB patients and 13 ALND patients, whose L-Dex scores were considered normal but showed an increasing trend coincidently with self-report of minor symptoms, were treated conservatively with a compression sleeve with no patient progressing to BCRL at last follow-up. Nine (seven ALND and two SLNB) patients had their L-Dex score resolve spontaneously without treatment (mean time to positive assessment = 8.2 months, mean time from positive assessment to resolution = 4.4 months). Further analysis of these subjects shows that for eight of these patients, positive assessment occurred within 9.1 months of initial surgery (mean = 4.3 months) and was resolved by the next visit (within 12.6 months, mean 3.0 months).

## Discussion

The results of this study demonstrate several key findings: (1) L-Dex was incorporated into routine breast cancer clinical practice and was used as part of routine follow-up care, (2) L-Dex scores identified patients in need of BCRL intervention, and (3) L-Dex was able to identify an improvement in BCRL following treatment. The incidence of BCRL for all patients in the present study, regardless of treatment type, was 12.3% (40 patients, mean follow-up time = 21.0 months). The incidence rates reported in the literature for all cancer treatments varies from 0 to 94%. These rates are primarily based on clinically evident BCRL measurable by tape circumference or water displacement. The cumulative incidence of BCRL in this cohort is lower than that expected from the literature (5). Additionally, the end of study incidence rates for this analysis (taking into consideration patients whose BCRL resolved during the study) have also shown to be much lower than chronic incidence rates reported in published studies with comparable follow-up durations. One SLNB patient (0.5%) (total follow-up time = 15.7 months) and 10 ALND patients (8.6%) (mean total follow-up time = 19.6 months) were assessed as persistent clinical BCRL at the end of the study. Comparable published studies reporting incidence rates with a similar duration of follow-up range from 3.5 to 16.8% for SLNB and 38.7 to 47.1% for ALND (see Table 1).

The changing management paradigm for BCRL is based on earlier detection and early intervention in order to prevent the chronic sequelae of BCRL that are irreversible (3, 5). As such, diagnostic modalities such as BIS allow for early detection by assessing the ECF and detecting subclinical increases (17, 18). Additionally, BIS as a diagnostic technique is objective with minimal inter- and intra-observer variability as compared with other techniques (28). With evidence-based guidelines supporting such a paradigm (27), trials are underway evaluating this approach; however, in the interim, the current data support continued study and utilization of early detection and treatment models. A large, prospective randomized trial evaluating L-Dex vs. circumference measurements with early intervention is currently accruing with results expected in the years to come.

The low chronic incidence rates demonstrated in the present study suggest that early detection is integral in the management of patients at risk of BCRL. By comparing ALND patients monitored with L-Dex technology with a control group monitored with tape measurements only, Soran et al. showed the importance of using L-Dex technology in early subclinical detection of BCRL and early intervention (26). The incidence of clinically apparent BCRL for patients in the control group was 36.4 and 4.4% for patients monitored with L-Dex technology. This suggests that prospective monitoring and treatment of subclinical BCRL using L-Dex technology can lead to reduced development of clinical BCRL. This study was limited by low patient numbers and lack of randomization. Moving forward, a randomized trial (as mentioned above) is currently underway, examining the role of BIS in allowing for early detection and subsequently early intervention with easily applied compression garments in order to prevent progression to complex decongestive physiotherapy (29).

Our result demonstrated that a subset of patients had spontaneous resolution of BCRL without treatment. This is consistent with Kilbreath et al. who found swelling in the first year after breast cancer treatment could possibly be transient due to the effects of exercise, surgery, anesthesia, and/or taxane therapy (30). Currently, there is limited data available to help differentiate those patients with transient increases in L-Dex and those who have persistent elevations, though future prospective studies will better address this.

There are limitations to the current analysis. This was a retrospective review and therefore, subject to the limitations of such an analysis. While the initial cohort was large, due to the small number of events, further data are required to validate these findings. Additionally, we were unable to evaluate other factors associated with an increased risk of lymphedema (i.e., radiation therapy, BMI) due to limits on the data available. Finally, because intervention was based on clinician discretion, no cut point for beginning intervention could be determined at this time. However, this study represents one of the few studies available that demonstrate the ability to use L-Dex as part of routine clinical breast care to identify subclinical BCRL and allow early intervention to prevent long-term chronic BCRL.

## Conclusion

The results of this retrospective study demonstrate that L-Dex assessments can be incorporated into routine breast cancer programs as part of follow-up. This is critically important given the recent changes in the NCCN survivorship guidelines for post-treatment follow-up care for breast cancer patients establishing that health-care providers “educate, monitor, and refer for lymphedema management.” Additionally, the analyses suggest that L-Dex assessments can identify subclinical BCRL and subsequently monitor the return to baseline following conservative interventions. Further studies are required to demonstrate the long-term benefits of early detection and subsequently early intervention predicated upon subclinical detection of BCRL.

## Author Contributions

AL contributed a portion of the retrospective patient data from her patient pool, analyzed all data, formulated a hypothesis, and was the primary author of this manuscript. BA contributed the remaining retrospective patient data from her patient population, assisted in formulating the hypothesis, and contributed significantly to the writing of this manuscript.

## Conflict of Interest Statement

The authors declare that the research was conducted in the absence of any commercial or financial relationships that could be construed as a potential conflict of interest.
